# Das Kaposi-Sarkom – eine Komplikation bei therapierefraktärer Colitis ulcerosa

**DOI:** 10.1007/s00292-022-01090-4

**Published:** 2022-06-24

**Authors:** Anne Kristin Fischer, Anton Kroesen, Reinhard Büttner, Uta Drebber

**Affiliations:** 1 Institut für Pathologie, Universität zu Köln, Kerpener Str. 62, 50937 Köln, Deutschland; 2 Krankenhaus Porz am Rhein, Köln, Deutschland

**Keywords:** Chronisch-entzündliche Darmerkrankung (CED), Immunmodulation, T-Zell-Suppression, HIV-unabhängiges Kaposi-Sarkom, α4β7-Integrin-Inhibitor, Chronic inflammatory bowel disease, Immunomodulation, T cell suppression, HIV-independent Kapos sarcoma, α4β7-Integrin inhibitor

## Abstract

Wir berichten über den Zufallsbefund eines Kaposi-Sarkoms des Kolons bei schwerer therapierefraktärer Colitis ulcerosa. Die Patientin war zuvor lange immunsuppressiv mit Infliximab, Vedolizumab und Prednisolon behandelt worden. Serologische Untersuchungen schlossen eine HIV(„human immunodeficiency virus“)-Infektion aus.

## Abstract (en)

We report on the incidental finding of Kaposi sarcoma of the colon in the setting of refractory ulcerative colitis treatment. The patient was under long-term immunosuppression with infliximab, vedolizumab, and prednisolone. Serologic analysis excluded human immunodeficiency virus (HIV) infection.

## Anamnese

Wir berichten über eine 45-jährige türkischstämmige Patientin mit 2019 erstdiagnostizierter Colitis ulcerosa. Im Januar 2021 zeigte die Koloskopie bis 80 cm ab ano eine floride (aktive) Entzündung. Die Patientin wurde nach refraktärer First-line-Therapie über mehrere Monate immunsuppressiv mit Vedolizumab, Infliximab und Prednisolon behandelt. Da auch diese Behandlung nicht anschlug, erfolgte eine restaurative Proktokolektomie. Zum Zeitpunkt der Operation erhielt sie lediglich 20 mg Prednisolon pro Tag.

## Pathologischer Befund

**Figure 1:**
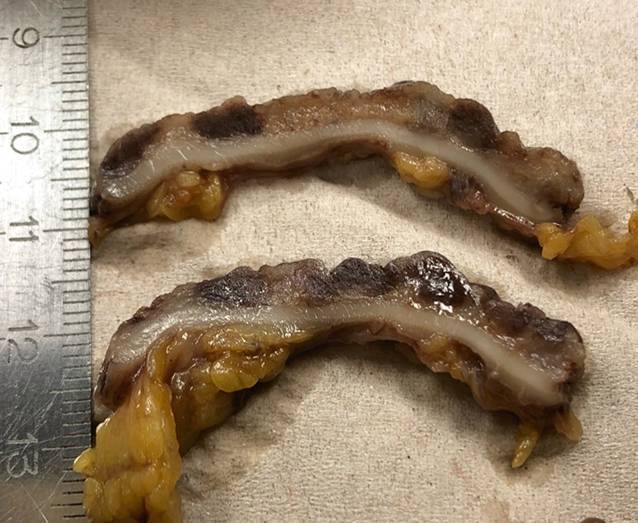

Das 64 cm lange Proktokolektomiepräparat zeigte eine starke ulzeröse Entzündung mit Granulationspolypen, darüber hinaus zahlreiche, über das gesamte Präparat verteilte, bis 7 mm messende schwerpunktmäßig submukosale rötlich-bräunliche, recht scharf umgrenzte Knoten (Abb. 1).

**Figure 2:**
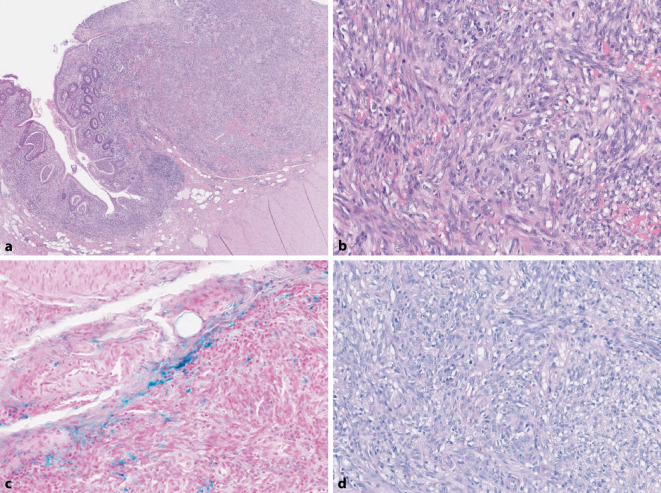

**Figure 3:**
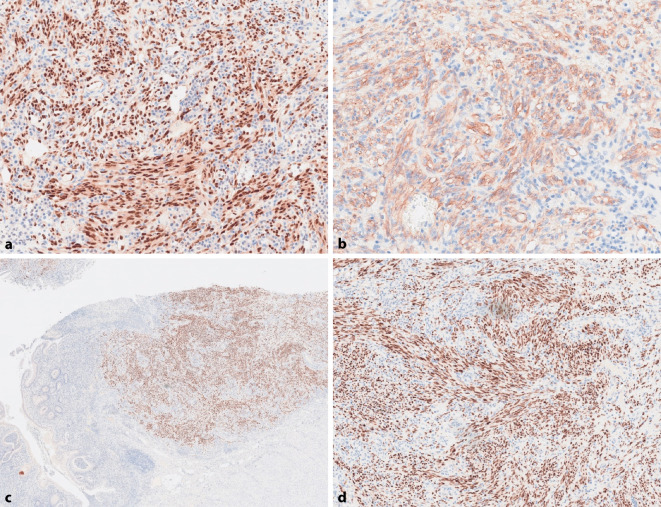

Histologisch bot sich das Bild einer aktiven Colitis ulcerosa mit Kryptenumbau und Kryptendestruktion ohne Dysplasien. Die mukosalen und submukosalen Herde erwiesen sich als spindelzellige, angedeutet faszikuläre vasoformative Tumoren mit schlitzartigen blutgefüllten Spalträumen. Dazwischen war fokal Siderin abgelagert. Ganz fokal fanden sich zwischen Endothelzellen und Basalmembran globuläre eosinophile Ablagerungen, hervorgehoben in der PAS-Färbung (Abb. 2a–d). Die endotheliale Natur der Proliferate bestätigte sich anhand ihrer CD34- und ERG-Expression (Abb. 3a, b). Die vasoformativen Endothel- und Spindelzellproliferate exprimierten intensiv nukleär das „long-acting nuclear antigene 1“ (LANA1) des HHV8 (Abb. 3c, d). Eine Zytomegalieinfektion konnte immunhistochemisch ausgeschlossen werden.

## Diagnose

Zusammengefasst handelt es sich somit um ein multifokales HHV8-positives Kaposi-Sarkom des Kolons bei über 24 Monate bestehender therapierefraktärer Colitis ulcerosa, einer iatrogen induzierten Form zuzuordnen aufgrund therapeutischer Immunsuppression unter anderem mit Vedolizumab.

## Verlauf

Im weiteren Verlauf ergab das bildmorphologische Staging mittels CT keine Hinweise auf Metastasen. Auch die angeschlossene Gastroskopie erwies sich als unauffällig. Serologische Untersuchungen schlossen eine HIV-Infektion der Patientin aus. Sechs Monate postoperativ bestand ein stabiler Zustand, ohne den klinischen Verdacht auf ein Rezidiv oder eine Manifestation der Erkrankung andernorts.

## Diskussion

Bei Kolektomiepräparaten nach langjähriger therapierefraktärer Colitis ulcerosa steht zunächst die makroskopische und histopathologische Begutachtung auf (a) das Befallsmuster, (b) die Entzündungsaktivität und (c) die Prüfung auf Dysplasien und hieraus entwickelten Karzinomen im Vordergrund. Einen zusätzlichen Aspekt bieten besondere therapieassoziierte Effekte, die durch neue spezifische immunmodulierende oder immunsupprimierende Antikörpertherapien mit unterschiedlichen Wirkstoffklassen im Rahmen einer lokalisierten oder systemischen Immunsuppression hervorgerufen werden (Tab. 1). Wie unsere Kasuistik und Literaturrecherche zeigen, ist hierbei auf besondere Malignommanifestationen zu achten, für die die antikörperbasierte immunsuppressive Behandlung verantwortlich zu machen sind.

**Table 1:** 

Wirkstoff	Gruppe	Wirkweise	Fälle	Patientenalter	Geschlecht	HIV	Zitat
Tofacitinib	Januskinaseinhibitor	Spezifischer Inhibitor der Januskinasen (v. a. JAK1 und JAK3)	1	30	m	Nein	Wetwittayakhlang P et al. 2021 [16]
Vedolizumab	Humanisierter monoklonaler IgG-G1-Antikörper gegen Integrin α4β7	„Hochselektive Blockade des Adhäsionsmoleküls α_4_β_7_-Integrin („lymphocyte Peyer’s patch adhesion molecule 1: LPAM-1) auf der Oberfläche aktivierter Lymphozyten, insbesondere auf Th-Gedächtniszellen. Hierüber wird die Bindung der Lymphozyten an MAdCAM-Rezeptoren auf dem Endothel von intestinalen Blutgefäßen und deren konsekutive Einwanderung in das Gewebe verhindert, die Entzündungsreaktion supprimiert“	3	45 47 30	w m m	Nein Ja Nein	Papa V et al. 2020 [12] Ajao SO 2021 [2] Fischer et al. 2022 [17]
Prednisolon	Kortikosteroide	Bindung des intrazellulären Glukokortikoidrezeptors, der in den Zellkern transloziert wird. Der Rezeptor-Liganden-Komplex bindet dann an spezifische Glukokortikoid-response-Elemente der DNA und aktiviert Gene, die Glukokortikoidantworten vermitteln	7	64 70 21 63 22 63 48	m m m m m m m	Nein Nein Nein N. b. Nein Nein Nein	Kumar V et al. 2017 [18] Hamzaoui L et al. 2013 [19] Adlersberg R 1969 [20] Thompson GB et al. 1989 [21] Du et al. 2018 [22]
Ciclosporin	Calcineurin-Inhibitor	Zyklisches Polypeptid aus 11 Aminosäuren (produziert durch die Pilzspezies Beauveria nivea). Calcineurin-Inhibitor. Stark immunsuppressive Eigenschaften insbesondere auf T‑Zellen und die zelluläre Immunantwort. Calcineurin aktiviert einen wichtigen Signaltransduktionsweg der T‑Zellaktivierung. Folge ist die vermindere der Reifung von T‑Lymphozyten und die verminderte Lymphokin-Produktion, einschließlich IL‑2	1	26	m	Nein	Clarke LM et al. 2021 [4]
Adalimumab	TNFα-Inhibitor	Monoklonaler rekombinanter IgG1-Antikörper gegen TNFα mit Bindung an den serum- und gewebegebundenen Tumornekrosefaktor, mit der Folge von dessen Inaktivierung und Degradation. Inhibition der Aktivität von TNFα führt zu einer Modulation des TNFα-vermittelten Entzündungs- und Schmerzpfades	1	70	m	Nein	Kumar V et al. 2017 [18] Hamzaoui L et al. 2013 [19]
Infliximab							
Golimumab							
Azathioprin/6-Mercaptopurin	Imidazol-Derivat (atypisches Nukleosid)	Nicht-kompetitiver Purin-Antagonist. Inhibition der Reifung von T‑Zellen, Blockade von Hypersensitivitätsreaktionen, antiinflammatorische Aktivität. Inhibition der Purin-Biosynthese und Inkorporation von 2‑Deoxy-6-Thioguanin-Nukleotiden in die die DNA. Die Zytotoxizität durch die Thiopurin-Inkorporation scheint die Methylierung einer Thiol-Gruppe zu erfordern, eine Fehlpaarung von S‑Methylguanin mit Thymin, mit folglicher Erkennung und Signalgebung an das Mismatch-repair-System	4	30 65 48 36	m m m w	Nein Nein Nein Nein	Hamzaoui L et al. 2013 [19] Rodríguez-Peláez M et al. 2010 [23] Du et al. 2018 [22] Puy-Montbrun et al. 1991 [24]
Mesalazin/Sulfasalazin	Modifiziertes Sulfonamid (Sulfapyridin und 5‑Aminosalizylsäure [5-ASA])	Inhibition des Arachidonsäuremetabolismus. Immunsuppression durch Hemmung der Zytokin-Synthese (IL‑1, IL‑2, TNFα, NFκB). Hierdurch mutmaßliche Inhibition der DNA-Synthese und Proliferation potenziell pathogener T‑ und B‑Zellpopulationen, Inhibition von Adhäsion, Chemotaxis und Phagozytose	1	49	m	Nein	Shah N et al. 2017 [25]

*N. b.* nicht bekannt

Das Kaposi-Sarkom ist ein angioproliferativer, HHV8-assoziierter Tumor [9]. Ursächlich ist insbesondere der Befall von Endothelzellen durch das HHV8-Virus, einem doppelsträngigen DNA-Virus [1, 3]. Vier Unterformen sind bekannt [1, 3, 6]:

- die klassische/sporadische Form,
- die endemische/afrikanische Form,
- die iatrogene und
- die AIDS-assoziierte Form.

Die *klassische/sporadische Form *wird v. a. bei über 50-jährigen Männern aus Europa oder dem mediterranen Raum beobachtet, sie manifestiert sich prädominant an den unteren Extremitäten.

Die *endemische/afrikanische Form* ist nicht HIV-assoziiert und findet sich bei jungen meist männlichen Erwachsenen zwischen 25 und 40 Jahren [15] und chronisch kranken oder immunsupprimierten kleinen Kindern im subsaharischen Afrika [11]; auch sie manifestiert sich an den unteren Extremitäten und kann bei Kindern aggressiv mit Lymphknotenmetastasen verlaufen.

Die *iatrogene Form* ist meist transplantationsassoziiert und befällt Haut, Schleimhäute und viszerale Organe [13, 14].

Die *AIDS-assoziierte/epidemische Form* letztendlich betrifft HIV1-infizierte, meist homosexuelle Männer, tritt bevorzugt in Haut und Schleimhäuten auf, kann einen disseminierten Befall zeigen und verläuft meist aggressiv. In diesem Zusammenhang entstehen die Tumoren im Stadium der Immuninsuffizienz mit einer CD4+-T-Zellzahl < 200/mm^3^ [3, 7].

In den westlichen Industrienationen ist ein HIV-unabhängiges Kaposi-Sarkom sehr ungewöhnlich. Bekannt sind transplantationsassoziierte Fälle, insbesondere nach Nierentransplantation [13]. In der englischsprachigen Literatur wird zunehmend über HIV-negative gastrointestinale Kaposi-Sarkome bei therapierefraktärer Colitis ulcerosa berichtet. Einschließlich der eigenen Beobachtung wurden unseres Wissens nach insgesamt 30 Fälle publiziert [4, 8, 10, 12]. Bei unterschiedlichen autoimmunen Grunderkrankungen, so bei Colitis ulcerosa, Morbus Crohn, rheumatoider Arthritis, Psoriasis oder Psoriasisarthritis scheint die Therapie mit einer Reihe von Immunsuppressiva oder Immunmodulatoren als seltene Komplikation die Entstehung eines Kaposi-Sarkoms zu begünstigen. Bemerkenswerterweise handelte sich hierbei jeweils um eine aktive bzw. therapierefraktäre Entzündung. In nahezu allen Fällen wurde das Kaposi-Sarkom als Zufallsbefund erst im Operationspräparat festgestellt. In diesem Zusammenhang wurden einschließlich dieses Berichts 3 Fälle eines nicht HIV-assoziierten Kaposi-Sarkoms nach Gabe von Vedolizumab publiziert, eines seit 2018 für schwere threapierefraktäre Colitis ulcerosa oder Morbus Crohn zugelassenen Integrinanatagonisten [4, 12]. Integrinantagonisten blockieren hochselektiv das Adhäsionsmolekül α_4_β_7_-Integrin auf der Oberfläche aktivierter T‑Lymphozyten. Hierüber wird die Bindung der T‑Lymphozyten an mukosale Addressin-Zell-Adhäsionsmolekül-1(MAdCAM1)-Rezeptoren auf dem Endothel intestinaler Blutgefäße und deren konsekutive Einwanderung in das Gewebe verhindert. Es kommt zu einer Unterdrückung des weiteren Entzündungsprozesses, folglich aber auch zu einer verminderten T‑Zell-vermittelten Immunantwort im Gastrointestinaltrakt. Andere, ebenfalls kontrovers diskutierte mögliche Auslöser eines Kaposi-Sarkoms sind Wirkstoffe aus der Gruppe der Januskinaseinhibitoren wie Tofacitinib [16]. Als weitere Gruppe kommen TNFα-Inhibitoren wie Adalimumab infrage, unter deren Einnahme auch das Auftreten anderer Malignome wie insbesondere maligner Melanome der Haut beschrieben werden.

Im Vergleich zum Kaposi-Sarkom bei systemischer HIV-Infektion ist die enterale Manifestation dieser Tumorentität bei chronisch entzündlicher Darmerkrankung unter der Gabe eines selektiv die T‑Zellen der Kolonschleimhaut unterdrückenden Immunsuppressivums bemerkenswert. Hier scheint offensichtlich das lokale Milieu gekennzeichnet durch Entzündung und therapeutische Modulierung bzw. Suppression von T‑Zell-Funktionen für die Tumorentstehung entscheidend zu sein. Dies erklärt auch, dass das alleinige Absetzen der Immunsuppressiva häufig schon zu einer Tumorremission führt. [16]. Medikamentös spricht das gastrointestinal manifeste Kaposi-Sarkom sehr gut auf eine Therapie mit pegyliertem liposomalem Doxorubicin und Rituximab an [5]. Bei gleichzeitiger schwerer Colitis ulcerosa mit notwendiger Immunsuppression muss eine (sub)totale Kolektomie erwogen werden.

Für andere Colitis-ulcerosa-Medikamente mit direkter T‑Zell-Suppression wird in der Literatur ebenfalls das Auftreten eines therapieassoziierten iatrogenen Kaposi-Sarkoms beschrieben. Zu nennen ist hier das Thiopurin Azathioprin, das über eine Störung der Purinbiosynthese und konsekutive DNA- und RNA-Bildungshemmung die Reifung von T‑Lymphozyten beeinträchtigt und somit Hypersensitivitätsreaktionen blockiert.

Auch unter Calcineurin-Inhibitoren-Therapie wie Tacrolimus oder Ciclosporin A wird das Auftreten iatrogener Kaposi-Sarkome berichtet, so insbesondere bei nierentransplantierten Patienten aber auch bei Colitis ulcerosa [4, 13].

## Fazit für die Praxis

- Bei einer immunsuppressiv langzeittherapierten Colitis ulcerosa mit immunmodulierenden oder -supprimierenden Medikamenten sollte auf die Möglichkeit der Entstehung eines iatrogenen HHV8-assoziierten Kaposi-Sarkoms geachtet werden.
- Bei Vorliegen atypischer Gefäßproliferate/bei histomorphologischem Verdacht auf Kaposi-Sarkom/spindelzelligem Sarkom sollte die immunhistochemische Untersuchung auf Expression des „long-acting nuclear antigene 1“ (LANA1) des HHV8 zielführend angeschlossen werden.
